# Perforated Meckel’s Diverticulum Masquerading as a Mesenteric Abscess Related to Umbilical Piercing: An Unusual Cause of Acute Abdomen

**DOI:** 10.7759/cureus.4020

**Published:** 2019-02-05

**Authors:** Jeeban Paul Das, Eoin O' Malley, Asif Iqbal, Clare Roche

**Affiliations:** 1 Radiology, University Hospital Galway, Galway, IRL; 2 Surgery, University Hospital Galway, Galway, IRL

**Keywords:** meckel’s diverticulum, umbilical piercing, belly-button piercing, mesenteric abscess, perforated meckel's diverticulitis

## Abstract

Perforated Meckel’s diverticulum (MD) is a rare cause of acute abdomen in adults. We describe the case of a 32-year-old man presenting with worsening abdominal pain three weeks following the piercing of his umbilicus. Computed tomography of the abdomen and pelvis demonstrated a small mesenteric collection intimately related to the recently placed navel ornamentation, and a preliminary diagnosis of intra-abdominal abscess secondary to an infected umbilical piercing was made. Initial conservative management with antibiotic therapy was unsuccessful. Subsequent open surgical approach demonstrated an inflamed, perforated Meckel’s diverticulum with a small, adjacent infected collection separate from, but in close proximity to the belly-button foreign body. The patient was successfully treated with small bowel resection and followed an uneventful postoperative course.

## Introduction

Meckel's diverticulum (MD) has an estimated incidence of 2% but complications related to the eponymous gastrointestinal tract diverticulum are rare, ranging from 2%-5% [1]. Perforation of MD can be life-threatening, and establishing the correct preoperative diagnosis may cause a diagnostic dilemma considering the overlap of clinical symptoms with other causes of acute abdominal pain, in addition to the often non-specific radiological findings on cross-sectional imaging [2, 3]. Umbilical piercings and navel ornamentation have increased in popularity and social acceptance in recent years. Infection related to piercings, however, is a well-recognized complication. This report details a case of a male in his 30s with a perforated Meckel’s diverticulum, who presented with acute abdominal pain, initially suspected as an infectious complication related to a recently placed umbilical piercing.

## Case presentation

**Figure 1:**
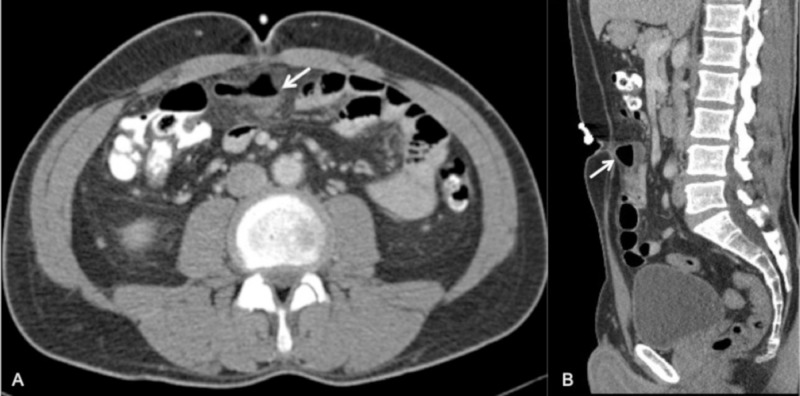
Axial (a) and sagittal (b) post-IV contrast-enhanced imaging demonstrating a gas- and fluid-filled mesenteric collection (arrows) located deep to the recently pierced umbilicus. IV - Intravenous, CT - computed tomography

A 32-year-old male with no prior medical or surgical history presented emergently with lower abdominal pain, nausea, and vomiting worsening over a two-day period. A physical exam revealed peri-umbilical tenderness with no abdominal distension, guarding, or tender rebound. Of note, an umbilical piercing was present, placed three weeks prior to presentation, surrounded by minor skin erythema. A full blood count revealed an elevated white cell count of 16.5 x 10^3^/uL with a relative neutrophilia of 78%. Normal liver function tests, electrolytes, and amylase were noted. Contrast-enhanced computed tomography (CT) of the abdomen and pelvis was performed, demonstrating a small (2.5 cm) air and fluid-filled collection deep to the umbilicus and extending between a small bowel loop (Figure 1).

**Figure 2:**
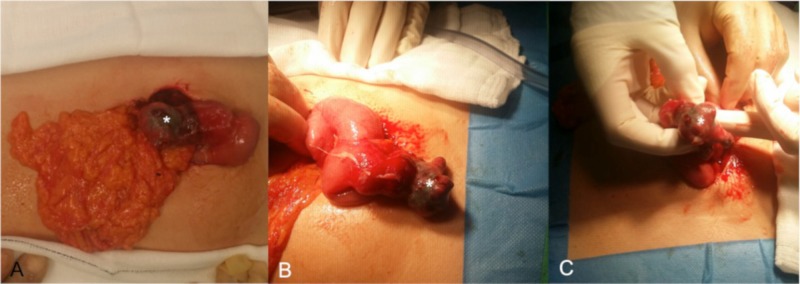
Intraoperative images (A-C) showing a blind-ending structure containing a small defect (*) arising from the distal ileum in keeping with a perforated inflamed Meckel's diverticulum.

The appendix appeared normal on CT and a preliminary diagnosis of an infected collection (related to the recently placed umbilicus ornamentation) with intra-abdominal extension was made. The patient was admitted to a general surgical ward and commenced on intravenous (IV) piperacillin and tazobactam, IV fluids, and he was kept nil by mouth. Following 24 hours of conservative management, the patient deteriorated clinically with fever, hypotension, tachycardia, and worsening abdominal pain, now radiating to the right iliac fossa. Blood cultures were drawn and the patient underwent an open surgical intervention. The caecum was identified and appeared normal. The appendix was noted in a retrocecal position and also appeared normal. Following identification of the terminal ileum, the distal ileum was then carefully examined in a retrograde fashion until a blind-ending, inflamed Meckel’s diverticulum was encountered, arising approximately 50 cm from the ileocecal valve proximally (Figure 2).

The purulent mesenteric collection identified on CT was also noted, lying deep to the umbilicus but separate from the navel piercing and extending between loops of distal ileum. These findings suggested a perforated Meckel’s diverticulum, rather than an infectious complication related to the recently placed body ornamentation.

A small bowel resection with functional end-to-end anastomosis was successfully undertaken and the surgical site thoroughly irrigated with normal saline solution. The remainder of the small bowel was unremarkable in appearance with no evidence of bowel ischemia.

The post-surgical period was uncomplicated and the patient was discharged on the third postoperative day. He remained well at clinical follow-up eight weeks after the date of his surgery.

Histopathological assessment of the resected specimen of the bowel confirmed MD, the surgical specimen demonstrating features of chronic, transmural ulceration with a small pocket of heterotopic gastric mucosa.

## Discussion

Meckel’s diverticulum, the most common malformation of the gastrointestinal (GI) tract, is a remnant of the prenatal vitelline duct that normally regresses between the fifth and seventh week of fetal life [1]. First described in 1809 by the eponymous anatomist [4], MD is a ‘true’ diverticulum, usually arising from the anti-mesenteric border of the small bowel [3, 5]. With an overall incidence of 2%, MD has a three times greater male preponderance toward symptomatic presentation [6]. Although most commonly presenting in people between one and four years of age, a second peak for presentation has been reported between seven and 16 years of age [7]. The risk of complication related to MD decreases with increasing age, with symptomatic cases rarely occurring in the adult population.

Much like the appendix, MD is also supplied by the superior mesenteric artery and is similarly vulnerable to infection, inflammation, and obstruction [8]. In addition, MD can contain ectopic gastric or pancreatic cells in 33%-50% of symptomatic cases, which may contribute to chronic inflammation of the blind-ending pouch, causing ulceration and risking perforation [6, 9].

Complications related to an MD include lower-gastrointestinal bleeding, abdominal pain, or bowel obstruction. Meckel’s diverticulitis can be seen in up to 20% of symptomatic adult patients with clinical signs often mimicking acute appendicitis [9]. Hemorrhage is the most common presentation of MD occurring in young children, reported in over 50% of cases. Bowel obstruction related to MD has been reported as the most common adult complication [10, 11]. Perforated Meckel’s diverticulum, however, is considered the most rarely encountered complication, occurring in less than 0.5% of symptomatic MD [12].

Establishing the correct preoperative diagnosis can be challenging considering the multitude of non-specific symptoms with which symptomatic MD can present. Complicated MD may be often misinterpreted as acute appendicitis with less than 10% of symptomatic cases of MD successfully diagnosed preoperatively [3].

**Figure 3:**
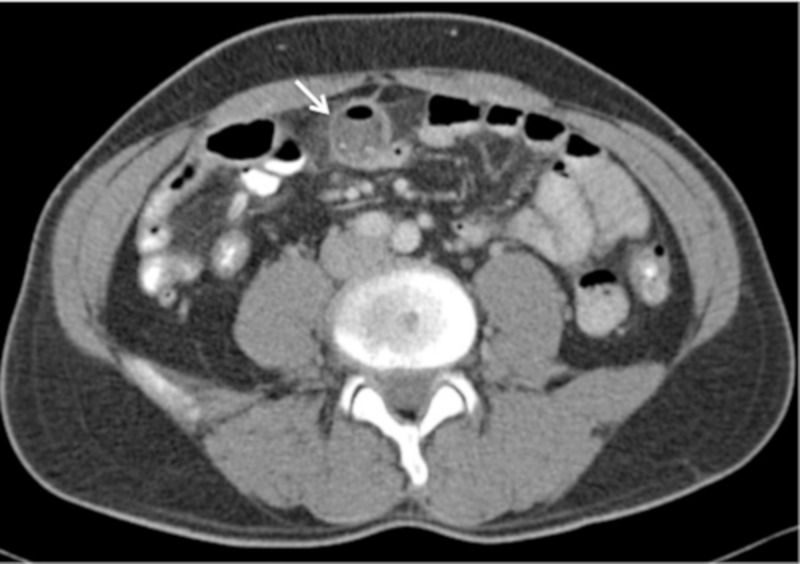
Retrospective review of axial post-IV contrast-enhanced CT imaging revealed a focal out-pouching of the distal ileum (arrow), just inferior and contiguous with the mesenteric collection, containing gas, fluid, and particulate matter with adjacent inflammatory change suggesting a perforated, inflamed Meckel's diverticulum. IV - intravenous, CT - computed tomography

In addition to a vague clinical presentation, the radiological features of complications related to Meckel’s diverticulum are frequently non-specific on cross-sectional CT [5]. Inflammation of Meckel's diverticulum usually appears as a blind-ending structure containing air, fluid and/or dependent material with mural thickening and surrounding mesenteric stranding [5]. A localized perforation can be suggested by the presence of air located outside of the bowel lumen or by an adjacent gas and fluid-containing collection [2, 5], as in this report. On retrospective review of the CT imaging in our case, a blind-ending pouch arising from the distal ileum was noted (Figure 3), containing fluid and particulate matter related to the fluid collection inferiorly, with adjacent mesenteric inflammatory change consistent with the postoperative diagnosis of perforated Meckel's diverticulum.

Nuclear medicine imaging using technetium-99m (Te-99m) pertechnetate radio-tracer offers an additional non-invasive diagnostic test to assist in identifying MD, providing a sensitivity and specificity of 97% and 94%, respectively [2, 5]. The radio-tracer is taken up by ectopic gastric mucosa, so in cases where heterotopic mucosa is absent, the nuclear medicine test will yield a negative result. Additionally, where there is no history of gastrointestinal bleeding, a higher rate of false negative Te-99m scans has been reported [1, 2].

The ‘rule of twos’ is a useful aide-memoire when considering the clinical, radiological, surgical, and histopathological findings of MD: occurring in 2% of the population, presenting before the age of two years, 2 inches long and located 2 feet from the ileocecal valve, and containing two types of heterotopic mucosa (gastric and pancreatic). Diverticular length and base diameter are well established factors predisposing to complications. The longer and more narrow-based diverticulum is considered more prone to obstruction and inflammation [1, 6, 10]. Diverticular morphology may also influence surgical treatment options, with diverticulectomy considered a safe and effective surgical option in long Meckel’s diverticulae. In patients with a sessile or short perforated MD, however, small bowel resection and anastomosis may be considered a more appropriate procedural alternative.

Umbilical piercings and navel ornamentation have increased in popularity in recent years. Infection related to piercings, however, is a well-recognized complication [13, 14]. The peri-umbilical area is a popular site of ornamentation and decoration with metallic piercings. Despite their social acceptability, navel piercings still account for up to 40% of complications related to body-piercings, with friction related to overlying clothing possibly exacerbating infection risks [13]. Infection involving the umbilicus may spread along the remnant of the umbilical cord causing intra-abdominal infection or abscess formation, as was initially suspected in this report [13, 14].

In our case, although a reasonable differential, given the clinical presentation and initial radiological assessment, a mesenteric abscess related to the umbilical piercing was not the correct preoperative diagnosis. This report highlights the importance of including pathology related to Meckel’s diverticulum in unusual cases of peritonitis. In addition, this case emphasizes the need to scrutinize preoperative imaging with a critical eye to exclude the possibility of MD complications as the etiology of an unexplained acute abdominal presentation.

## Conclusions

A high index of clinical suspicion must be maintained towards the possibility of complicated MD in cases of acute abdomen, in particular when other abdominal pathology has been excluded. Detailed examination (and possibly re-analysis) of cross-sectional abdominal imaging to identify possible features of complicated MD in peritonitic patients should be considered where the preoperative diagnosis is equivocal.
